# Saturation Mutagenesis of Lysine 12 Leads to the Identification of Derivatives of Nisin A with Enhanced Antimicrobial Activity

**DOI:** 10.1371/journal.pone.0058530

**Published:** 2013-03-11

**Authors:** Evelyn M. Molloy, Des Field, Paula M. O'. Connor, Paul D. Cotter, Colin Hill, R. Paul Ross

**Affiliations:** 1 Department of Microbiology, University College Cork, Cork, Ireland; 2 Alimentary Pharmabiotic Centre, University College Cork, Cork, Ireland; 3 Teagasc Food Research Centre, Moorepark, Fermoy, Co. Cork, Ireland; University Medical Center Utrecht, The Netherlands

## Abstract

It is becoming increasingly apparent that innovations from the “golden age” of antibiotics are becoming ineffective, resulting in a pressing need for novel therapeutics. The bacteriocin family of antimicrobial peptides has attracted much attention in recent years as a source of potential alternatives. The most intensively studied bacteriocin is nisin, a broad spectrum lantibiotic that inhibits Gram-positive bacteria including important food pathogens and clinically relevant antibiotic resistant bacteria. Nisin is gene-encoded and, as such, is amenable to peptide bioengineering, facilitating the generation of novel derivatives that can be screened for desirable properties. It was to this end that we used a site-saturation mutagenesis approach to create a bank of producers of nisin A derivatives that differ with respect to the identity of residue 12 (normally lysine; K12). A number of these producers exhibited enhanced bioactivity and the nisin A K12A producer was deemed of greatest interest. Subsequent investigations with the purified antimicrobial highlighted the enhanced specific activity of this modified nisin against representative target strains from the genera *Streptococcus*, *Bacillus*, *Lactococcus, Enterococcus* and *Staphylococcus*.

## Introduction

Bacteriocins are small (<4 kDa) ribosomally-synthesised peptides secreted by bacteria into their environment and which inhibit other bacteria. They can have a broad (effective against multiple genera) or narrow (effective only against specific species) activity spectra. Producer organisms also have specific immunity proteins to protect them from the action of their own bacteriocin. Nisin is an example of the class I bacteriocins, distinguished by virtue of containing unusual amino acids as a result of enzyme-mediated post-translational modification [1]. This particular group of Class I are known as the lanthionine-containing **antibiotics** or ‘**lantibiotics**’ [2]. In lantibiotics, the unusual amino acids dehydroalanine (Dha) and dehydrobutyrine (Dhb) are formed by dehydration of serine and threonine residues, respectively. Subsequently, specific addition reactions between cysteine residues and some of these unsaturated amino acids result in the formation of the characteristic lanthionine and β-methyllanthionine residues. The thio-ether bridges of these residues act as intramolecular cross-links, resulting in the introduction of ‘rings’ in the mature bacteriocin [3], [4], [5].

**Figure 1:**
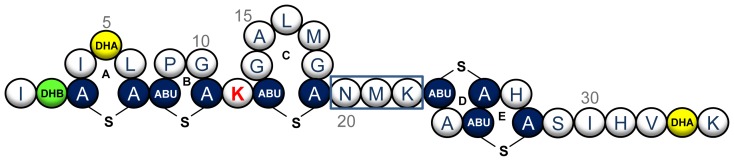
Structure of nisin A. The structure of nisin A is depicted, showing the location of its five (β-methyl)lanthionine rings (A–E) and modified residues dehydroalanine (yellow) and dehydrobutyrine (green). Position K12 is highlighted in red and the hinge region is boxed.

Six natural nisin variants have been identified to date, namely nisin A [6], nisin Z [7], nisin Q [8], nisin F [9] (produced by strains of *Lactococcus lactis*), and nisin U and nisin U2 (produced by strains of *Streptococcus uberis*) [10]. Nisin A contains 34 amino acids and five post-translationally incorporated (β-methyl)lanthionine rings (Fig. 1) [4]. It displays antibacterial activity against a wide range of Gram-positive bacteria, including food-borne pathogens such as staphylococci, bacilli and clostridia. Studies have revealed that nisin and several other lantibiotics use the membrane-bound peptidoglycan precursor lipid II as a docking molecule [11]. In the case of nisin, this facilitates two bactericidal activities, the inhibition of cell wall biosynthesis and disruption of the cell membrane due to pore formation [12], [13], [14]. This dual activity is possible due to the presence of two structured domains, both of which are amphipathic in character [15]. The N-terminal domain, containing rings A, B, and C, is linked to the C-terminal intertwined rings D and E by a flexible hinge region consisting of three residues (N20-M21-K22; Fig. 1). Structural studies have established that rings A and B form a ‘cage’ that facilitates binding of the pyrophosphate moiety of lipid II, thus interfering with cell wall synthesis [16]. This binding allows the C-terminal rings D and E, via the flexible hinge region, to form pores in the target membrane, causing cell death by rapid efflux of ions and cytoplasmic solutes [17], [18].

Although nisin has been studied extensively from a fundamental perspective, there has been an even greater focus on its commercial application. Nisin provides an excellent solution to consumer demands for safe food with a long shelf-life, but without the need for chemical preservatives. Nisin has a long record of safe use and is used in a variety of foods such as canned foods, processed cheese and dairy products [19], [20]. It has been approved by the EU as additive E234, as well as by the World Health Organisation (WHO) and the US Food and Drug Administration (FDA). In addition to food-related applications, nisin has been investigated as a chemotherapeutic agent given its high potency (with activity at nanomolar concentrations) [21], [22] and its stable, non-cytotoxic nature [23]. As nisin is active against the principle Gram-positive pathogens that cause bovine mastitis, an infection of the udder that is both persistent and costly to treat, it has also been incorporated into a number of commercial products as an efficacious and non-toxic alternative to antibiotics [24], [25], [26]. Finally, nisin shows promise in suppressing multi-drug resistant infections such as methicillin-resistant *Staphylococcus aureus* (MRSA), heterogeneous vancomycin-intermediate *S. aureus* (hVISA), and vancomycin-resistant *Enterococcus* (VRE) [22], [27].

Because lantibiotics are gene-encoded and ribosomally synthesized (as opposed to being assembled *via* multi-step, multi-enzyme processes as is the case for non-ribosomally synthesised peptide antibiotics), they are amenable to relatively simple bioengineering strategies designed to improve their production and effectiveness [4], [5], [28], [29]. Nisin has been the focus of bioengineering in order to elucidate the relationship between the structure and function of the molecule, as well as with a view to improving functional properties of the peptide [30]. Bioengineering has been fruitful with respect to the introduction of mutations that have a positive impact on the physico-chemical properties of nisin, including better solubility [31], [32], improved stability [32] and an enhanced ability to diffuse through complex polymers [33]. Initial success with regard to increasing the potency of nisin came with the creation of bioengineered nisin derivatives with superior antimicrobial activity against some non-pathogenic targets [13], [34], [35], [36]. Perhaps more importantly, nisin Z N20K and M21K were the first bioengineered nisin derivatives that were enhanced against pathogenic bacteria, namely *Shigella*, *Pseudomonas* and *Salmonella* species [32]. The first nisin derivatives to be created with improved activity against target Gram-positive pathogens were nisin A N20P, M21V, K22S and K22T [37]. Nisin M21V has since been designated as Nisin V and exhibits enhanced activity a wide range of targets, including numerous drug resistant strains [38]. Nisin V outperformed wild-type nisin in a food model with respect to the control of the food pathogen *Listeria monocytogenes*, a noteworthy outcome in light of the high innate resistance of *L. monocytogenes* to nisin [38]. Recently, nisin A S29G, S29A, S29D, S29E derivatives were described that displayed increased potency against a range of Gram-positive targets, with S29G and S29A being the first nisin derivatives found to display enhanced activity against both Gram-positive and Gram-negative bacteria [39]. Taken together, these studies demonstrate that bioengineering can both improve the activity of nisin against sensitive cells as well as alter its target spectrum.

Given the previous positive outcomes from manipulating the nisin ‘hinge’ region [32], [37], we turned our attentions to K12 of nisin A, a residue which could serve as a smaller flexible region between rings B and C (Fig. 1). A site-saturation mutagenesis approach in a *L. lactis* NZ9800 background led to the discovery of several derivatives of interest with one in particular, K12A, displaying enhanced specific activity against numerous Gram-positive microorganisms of food and/or clinical significance. This discovery further highlights the benefits of bioengineering nisin to generate derivatives with superior antimicrobial activity against specific problematic microorganisms.

## Materials and Methods

### Bacterial strains and growth conditions

Bacterial strains and plasmids used in this study are listed in Table 1. *L. lactis* strains were grown at 30°C in M17 broth (Oxoid) supplemented with 0.5% glucose (GM17) or on GM17 agar (1.5% w/v). *C. sakazakii*, *Salmonella* and *E. coli* strains were grown at 37°C in Luria-Bertani (LB) broth with vigorous shaking or on LB agar. Chloramphenicol was used at 10 µg/ml for *L. lactis* and *E. coli* where necessary. *Enterococcus* strains were grown at 37°C in GM17 broth or on GM17 agar. *Streptococcus* strains were grown in Tryptic Soy Broth (TSB) (Merck) or on TSB agar at 37°C (supplemented with 0.3% (w/v) yeast extract (Oxoid) in the case of *S. pyogenes* DSM 11728; grown microaerophilically), except *S. pyogenes* DSM 2071 which was grown anaerobically (Anaerocult A; Merck, Darmstadt, Germany) in Brain Heart Infusion (BHI) broth (Oxoid) or on BHI agar at 37°C. *Staphylococcus* and *Bacillus* strains were grown at 37°C in BHI broth or on BHI agar. Drug resistant strains (i.e. VREs, hVISAs and MRSAs) were grown in Mueller-Hinton (MH) broth (Oxoid) or on MH agar at 37°C. *Listeria* strains were grown in BHI broth or on BHI agar at 37°C.

**Table 1:** Strains and plasmids used in this study.

Strain/plasmids	Relevant characteristics	Source/reference
Strains		
*Lactococcus lactis* NZ9800	*L. lactis* NZ9700 Δ*nisA*	[63], [64]
*L. lactis* NZ9800 pDF05	Wild-type nisin A producer	[37]
*Escherichia coli* Top10	Intermediate cloning host	Invitrogen
Indicator Organisms		
*Enterococcus faecalis* 5152	Indicator strain	DPC Collection
*E. casseliflavus* 5053	Indicator strain	DPC Collection
*E. faecium* VRE EC 533	Indicator strain	BSAC
*E. faecium* VRE EC 725	Indicator strain	BSAC
*E. faecium* 5119	Indicator strain	DPC Collection
*E. faecium* 5137	Indicator strain	DPC Collection
*E. durans* 5133	Indicator strain	DPC Collection
*Streptococcus dysgalactiae* UCC 5003	Indicator strain	UCC Culture Collection
*S. dysgalactiae* GroupC #2	Indicator strain	UCC Culture Collection
*S. pyogenes* DSM 2071	Indicator strain	DSMZ
*S. pyogenes* DSM 11728	Indicator strain	DSMZ
*S. pyogenes* NCDO 2381	Indicator strain	NCDO
*S.agalactiae* ATCC 13813	Indicator strain	ATCC
*S.agalactiae* Group B	Indicator strain	UCC Culture Collection
*S.agalactiae* COH31rs	Indicator strain	DPC Collection
*S. mitis* UCC 5001	Indicator strain	UCC Culture Collection
*Bacillus cereus* NCIMB 700578	Indicator strain	NCIMB
*B. cereus* NCIMB 700579	Indicator strain	NCIMB
*B. cereus* NCIMB 700827	Indicator strain	NCIMB
*B. cereus* DPC 6089	Indicator strain	DPC Collection
*B. cereus* DPC 5334	Indicator strain	DPC Collection
*B. cereus* NCIMB 700577	Indicator strain	NCIMB
*B. subtilis* UCC 5002	Indicator strain	UCC Culture Collection
*L. lactis* ssp *cremoris* HP	Indicator strain	UCC Culture Collection
*L. lactis* MG 1363	Indicator strain	UCC Culture Collection
*Staphylococcus aureus* hVISA 32679	Indicator strain	BSAC
*S. aureus* hVISA 32652	Indicator strain	BSAC
*S. aureus* ST MRSA 534	Indicator strain	BSAC
*S. aureus* ST MRSA 528	Indicator strain	BSAC
*S. aureus* ST MRSA 530	Indicator strain	BSAC
*S. aureus* RF 122	Indicator strain	DPC Collection
*S. aureus* DPC 5247	Indicator strain	DPC Collection
*S. aureus* DPC 5971	Indicator strain	DPC Collection
*S. aureus* NCDO 1499	Indicator strain	DPC Collection
*Listeria monocytogenes* EGD-e	Indicator strain; serotype 1/2a	[65]
*L. monocytogenes* EGD-e Δ*virR*	Indicator strain; Δ*virR*	[51]
*L. monocytogenes* EGD-e Δ*telA*	Indicator strain; Δ*telA*	[66]
*L. monocytogenes* L028	Indicator strain; clinical isolate	UCC Culture Collection
*L. monocytogenes* L028 Δ*gadA*	Indicator strain; Δ*gadA*	[67]
*L. monocytogenes* L028.pORI.*lmo*1021	Indicator strain	Guinane et al., unpublished
*L. monocytogenes* 10403S	Indicator strain	UCC Culture Collection
*L. monocytogenes* 33410	Indicator strain; clinical isolate; serotype 4b	ILSI
*L. monocytogenes* 33423	Indicator strain; food isolate; serotype 1/2b	ARS
*Cronobacter sakazaki* DPC 6440	Gram-negative indicator strain	DPC Collection
*Salmonella enterica* serovar Typhimurium UK1	Gram-negative indicator strain	UCC Culture Collection
*E. coli* 0127∶H6	Gram-negative indicator strain	UCC Culture Collection
**Plasmids**		
pDF05	pCI372 with *nis*A under its own promoter	[37]
pDF05-K12A	pDF05 with K12A substitution (GCT) in *nisA*	This study

DPC: Dairy Products Research Centre, Moorepark; UCC: University College Cork; NCDO: National Collection of Dairy Organisms; DSMZ: German Collection of Microorganisms and Cell Cultures; ATCC: American Type Culture Collection; NCIMB: National Collection of Industrial, Food and Marine Bacteria; BSAC: British Society for Antimicrobial Chemotherapy; ILSI: International Life Science Institute (MartinWiedmann), ARS: Agricultural Research Service, U.S. Department of Agriculture (Todd Ward).

### Site-saturation and site-directed mutagenesis of position K12 of nisin A

Saturation mutagenesis of the lysine codon at position 12 of *nis*A was performed by PCR using plasmid pDF05 [37] as template (Table 1) and oligonucleotides NisK12degFOR and NisK12degREV (Table 2). These oligonucleotides contain an NNK codon in place of the native AAA codon, in theory resulting in the substitution of the native residue with all 19 other standard amino acids [40], [41]. PCR amplification was performed as previously described [37]. Following introduction into the *E. coli* Top10 intermediate host, plasmid DNA was isolated and sequenced (MWG Operon, Germany) with pCI372For (Table 2) to confirm mutagenesis of the specific codon. Plasmid DNA was introduced by electroporation into the expression strain *L. lactis* NZ9800. Approximately 150 transformants were subjected to colony mass spectrometry (CMS), which in most instances could be used to unequivocally determine the nature of the amino acid substitutions. DNA sequencing with pCI372For (Table 2) was employed in cases where further clarification was needed.

**Table 2:** Oligonucleotides used in this study.

Primer name	Sequence
NisK12degFOR	5′- PHO CCCGGTTGTNNKACAGG GCTCTGATGGGTTGTAACATG -3′
NisK12degREV	5′- AGCTCCTGTMNNACAACCGGGTGTACATAGCGAAATACT -3′
pCI372FOR	5′- CGGGAAGCTAGAGTAAGTAG -3′
pCI372Rev	5′- ACCTCTCGGTTATGAGTTAG -3′
K12HFor	5′- CCCGGTTGTcacACAGGAGCTCTGATGG -3′
K12HRev	5′- AGCTCCTGTgtgACAACCGGGTGTACATAGC -3′
K12Hcheck	5′- TATGTACACCCGGTTGTcac -3′
K12DFor	5′- CCCGGTTGTGacACAGGAGCTCTGATGG -3′
K12DRev	5′- AGCTCCTGTgtCACAACCGGGTGTACATAGC -3′
K12Dcheck	5′- TATGTACACCCGGTTGTGac -3′
K12NFor	5′- CCCGGTTGTaaTACAGGAGCTCTGATGG -3′
K12NRev	5′- AGCTCCTGTAttACAACCGGGTGTACATAGC -3′
K12Ncheck	5′- TATGTACACCCGGTTGTaa -3′
K12IFor	5′- CCCGGTTGTatcACAGGAGCTCTGATGG -3′
K12IRev	5′- CCCGGTTGTatcACAGGAGCTCTGATGG -3′
K12Icheck	5′- TATGTACACCCGGTTGTatc- 3′

PHO indicates 5′ phosphate modification. Underlined sequences represent degenerate codon (N = A+C+G+T, K = G+T, M = A+C). Lower-case letters indicate site-directed mutation.

Site-directed mutagenesis of the *nis*A gene was achieved using pDF05-K12A as template (Table 1), the relevant primers (Table 2) and PCR amplification as before. To detect altered Top10 transformants, candidates were screened by PCR using a specific ‘check’ primer and pCI372Rev (Table 2). Plasmids from candidates were sequenced using pCI372Rev (Table 2) to verify the deliberate mutation and to confirm no other changes had been introduced, then used to transform NZ9800.

### Deferred antagonism assays

Deferred antagonism assays for bioactivity determination were performed as previously described [39].

### Matrix-assisted laser desorption/ionization time-of-flight (MALDI-TOF) mass spectrometric analysis

Colony mass spectrometry (CMS was performed with an Axima TOF^2^ MALDI-TOF mass spectrometer (Shimadzu Biotech, Manchester, UK) in positive-ion reflectron mode as previously described [39]. In the case of purified peptide, a small amount of lyophilised peptide resuspended in 70% 2-propanol 0.1% triflouroacetic acid (TFA) was used for mass spectrometric (MS) analysis.

### Reverse phase-high performance liquid chromatography (RP-HPLC) purification of nisin and derivatives

Purified nisin A (nisin) and its derivatives were obtained in a modified version of a previously employed protocol [38]. The Phenomenex C12 RP-HPLC column (Jupiter 4 µ proteo 90 Å, 250 X 10.0 mm, 4 µm) was developed in a gradient of 30% acetonitrile (ACN) 0.1% TFA to 50% ACN containing 0.1% TFA over 5–40 min, at a flow rate of 1.8 ml/min. In order to facilitate a comparison of production levels, identical purification steps were employed and production quantified on the basis of peak areas obtained during RP-HPLC as calculated with Shimadzu class VP software (Shimadzu Biotech, Manchester, UK). The gradient for separation of K12S/Dha and K12T/Dhb was 35% ACN 0.1% TFA to 45% ACN 0.1% TFA over 5–40 min, at a flow rate of 1.8 ml/min. Fractions containing peptide were collected after each RP-HPLC run, pooled and the ACN removed by rotary evaporation. Lyophilised peptide was stored at −20°C.

### Minimum inhibitory concentration assays

Minimum inhibitory concentration (MIC) determinations were performed as previously described [39]. Wild-type nisin and nisin mutant peptides were adjusted to a starting concentration of 30 µM (*Enterococcus faecalis* and *casseliflavus*), 10 µM (*Streptococcus*, *Bacillus*, MRSA), 5 µM (*Enterococcus faecium* VRE) or 500 nM (*Lactococcus*) and two-fold serial dilutions of each peptide were added to the target strain. After incubation for 16 h under the relevant conditions, the MIC was read as the lowest peptide concentration causing inhibition of visible growth.

### Gram-negative agarose gel diffusion assay

Equimolar amounts of purified nisin A and K12A (40 µM) were assessed for bioactivity against representative Gram-negative species as previously described [39].

## Results

### Creation and mass spectrometric analysis of a bank of nisin K12 derivatives

A site-saturation mutagenesis-based strategy was undertaken to generate a bank of producers of bioengineered nisin derivatives in which residue K12 was converted to every other natural amino acid. This strategy proved successful in that 15 of the 19 potential residue conversions were identified. Site-directed mutagenesis was employed to create the remaining derivatives (K12N, K12I, K12D and K12H), resulting in the generation of a complete “K12X” bank of producers (Table 3). It is notable that the K12S and K12T substitutions introduce hydroxyl residues, which could potentially become substrates for the lanthionine modification machinery to produce Dha or Dhb, respectively. Analysis of these derivatives by CMS confirmed that modification occurs to some degree in that we detected masses corresponding to Dha and Dhb, in addition to masses indicating the presence of the unmodified residues (Table 3). In the case of the K12D derivative, a peptide of the corresponding mass could not be detected by CMS, indicating a detrimental impact on production. CMS analysis of the K12C derivative showed that the newly incorporated cysteine remains in an unmodified form.

**Table 3:** Mass Spectrometry Analysis and Bioactivity Determination of Nisin K12X Bank.

	Amino Acid		Molecular Mass		Bioactivity
	K12X		Predicted	Observed	Mean (S.D)
**Hydrophilic: Charged**	Lysine+ve	K	3353.06	3353.04	100 (N/A)
	Histidine+ve	H	3362.03	3361.40	**75** (5)
	Arginine+ve	R	3381.08	3381.24	**61** (6)
	Glutamic acid -ve	E	3354.01	3353.74	0 (N/A)
	Aspartic acid -ve	D	3339.98	ND	0 (N/A)
**Hydrophilic: Neutral**	Threonine	T	3326.00	3326.28	
					**129** (3)
			3307.98*	3307.63*	
	Serine	S	3311.98	3311.14	
					**128** (1)
			3293.95*	3293.65*	
	Asparagine	N	3339.00	3338.74	108 (9)
	Glutamine	Q	3353.03	3353.48	102 (9)
	Tyrosine	Y	3388.07	3388.43	**73** (7)
**Hydrophobic**	Alanine	A	3295.98	3296.49	**131** (2)
	Proline	P	3322.01	3321.71	**118** (7)
	Valine	V	3324.03	3323.76	107 (9)
	Methionine	M	3356.08	3355.55	105 (9)
	Cysteine	C	3328.04	3328.89	103 (8)
	Leucine	L	3338.05	3339.02	89 (5)
	Isoleucine	I	3338.05	3337.88	89 (9)
	Glycine	G	3281.94	3281.90	**68** (2)
	Tryptophan	W	3411.10	3410.83	**63** (5)
	Phenylalanine	F	3372.07	3372.16	**54** (5)

Observed molecular mass (+/−0.25 Da) from MALDI-TOF MS analysis of NZ9800 pDF05-K12X producers. ND: not detected. * represents dehydrated forms (hydrophobic modified residues), i.e. Dhb in the case of T and Dha in the case of S.

Bioactivity of NZ9800 pDF05-K12X producers against *L. lactis* HP. Values given are the mean of triplicate deferred antagonism assays and represent zone of inhibition (diameter of zone minus diameter of bacterial growth) expressed as a percentage compared to that of the wild-type nisin producer at 100%. S.D.: Standard Deviation; Relative Standard Deviation <10% for each given value. N/A: not applicable. All values in bold reached statistical significance compared to nisin control (K) (Student's t-test: P<0.05).

### Bioactivity of the nisin A K12X bank

The bioactivity of the 19 K12X derivatives was tested against a standard sensitive indicator strain (*L. lactis* HP) by deferred antagonism assays, with the sizes of the zones of inhibition being assessed relative to that of the corresponding wild-type nisin A producer (Table 3). Bioactivity reflects the overall activity of producer strains and does not discriminate between effects due to increased/decreased specific activity, altered peptide production levels, or effects on other physico-chemical properties such as diffusion in agar. Assaying for bioactivity provides a valuable initial screen to facilitate the identification of derivatives in which beneficial changes have occurred. Indeed, bioactivity was found to be significantly enhanced in the cases of K12A, K12S, K12T and K12P (Table 3).

### Bioactivity spectrum of lead nisin K12X derivatives

Because the nisin K12A, K12S and K12T producers had the highest bioactivity levels (>125% compared to the wild-type control), they were selected for further analysis. In order to gain an insight into target specificity, their bioactivity levels were assessed against 42 target strains corresponding to 13 species of food and/or clinical relevance (Table 4). Although bioactivity was significantly enhanced against most Gram-positive strains tested, it is important to note that the level of enhanced activity shown by each derivative was strain variable. In the case of *L. monocytogenes*, all displayed bioactivity equal to or less than the wild-type producer (Table 4), with the antimicrobial sensitive Δ*virR* mutant of *L. monocytogenes* EGDe being an exception to this general trend. While the K12A, K12S and K12T producers show a similar spectrum of bioactivity, the K12A derivative consistently showed the greatest bioactivity. Furthermore, significantly enhanced bioactivity for K12A was evident against every strain from 5 of the 6 genera tested, excepting *L. monocytogenes* as previously mentioned. The K12A, K12S and K12T producers were not active against the wild-type strain (data not shown).

**Table 4:** Bioactivity of K12A, K12S and K12T producers against various Gram-positive targets.

Strain		NisinK12A	NisinK12S	NisinK12T
*Enterococcus faecalis*	5152	**211** (19)	**164** (8)	**181** (3)
*Enterococcus casseliflavus*	5053	**174** (14)	**169** (15)	**166** (16)
*Enterococcus faecium*	VRE EC533*	**166** (2)	**150** (6)	**132** (11)
*Enterococcus faecium*	VRE EC725*	**143** (7)	**124** (6)	117 (8)
*Enterococcus faecium*	5119	**150** (9)	**131** (0)	117 (9)
*Enterococcus faecium*	5137	**140** (12)	**121** (5)	125 (11)
*Enterococcus durans*	5133	**127** (7)	**118** (4)	**121** (3)
*Streptococcus dysgalactiae*	UCC 5003	**175** (10)	**147** (15)	**136** (9)
*Streptococcus dysgalactiae*	GroupC #2	**145** (6)	**123** (5)	**113** (6)
*Streptococcus pyogenes*	DSM 2071	**170** (3)	**143** (4)	**137** (5)
*Streptococcus pyogenes*	DSM 11728	**132** (6)	**122** (8)	**119** (3)
*Streptococcus pyogenes*	NCDO 2381	**127** (10)	**115** (1)	**115** (3)
*Streptococcus agalactiae*	ATCC 13813	**142** (8)	**123** (8)	**120** (4)
*Streptococcus agalactiae*	GroupB	**134** (6)	**124** (7)	**127** (3)
*Streptococcus agalactiae*	COH31rs	**133** (6)	**124** (11)	**126** (11)
*Streptococcus mitis*	UCC 5001	**139** (4)	115 (8)	122 (10)
*Bacillus cereus*	NCIMB 700578	**167** (8)	**162** (7)	**158** (10)
*Bacillus cereus*	NCIMB 700579	**141** (3)	**135** (6)	124 (11)
*Bacillus cereus*	NCIMB 700827	**133** (10)	**120** (5)	117 (12)
*Bacillus cereus*	DPC 6089	**128** (10)	**120** (4)	**116** (4)
*Bacillus cereus*	DPC 5334	**126** (10)	110 (8)	105 (8)
*Bacillus cereus*	NCIMB 700577	**121** (10)	**118** (10)	**112** (6)
*Bacillus subtilis*	UCC 5002	**129** (7)	**112** (4)	**120** (7)
*Lactococcus lactis*	MG1363	**140** (3)	**128** (7)	**131** (7)
*Staphylococcus aureus*	hVISA 32679*	**140** (12)	**132** (7)	**124** (9)
*Staphylococcus aureus*	hVISA 32652*	**136** (3)	**134** (3)	**124** (5)
*Staphylococcus aureus*	MRSA ST 534*	**148** (9)	**140** (14)	**137** (10)
*Staphylococcus aureus*	MRSA ST 528*	**134** (6)	**127** (5)	**129** (7)
*Staphylococcus aureus*	MRSA ST 530*	**121** (1)	**107** (3)	**110** (1)
*Staphylococcus aureus*	RF 122	**130** (7)	**124** (5)	**120** (5)
*Staphylococcus aureus*	DPC 5247	**126** (3)	**119** (3)	**114** (5)
*Staphylococcus aureus*	DPC 5971	**124** (9)	**116** (2)	**111** (3)
*Staphylococcus aureus*	NCDO 1499	**111** (3)	109 (5)	**109** (3)
*Listeria monocytogenes*	EGD-e	106 (10)	97 (4)	100 (7)
*Listeria monocytogenes*	EGD-eΔ*virR*	**133** (8)	**122** (6)	**121** (9)
*Listeria monocytogenes*	EGD-eΔ*telA*	**93** (4)	**91** (5)	95 (7)
*Listeria monocytogenes*	L028	104 (6)	95 (5)	104 (10)
*Listeria monocytogenes*	L028Δ*gadA*	101 (9)	98 (8)	89 (7)
*Listeria monocytogenes*	L028 pORI19HK	99 (8)	102 (3)	106 (6)
*Listeria monocytogenes*	10403S	103 (8)	99 (9)	102 (5)
*Listeria monocytogenes*	33410	94 (7)	95 (9)	85 (8)
*Listeria monocytogenes*	33423	85 (6)	**89** (0)	**75** (6)

Values are the mean of triplicate deferred antagonism assays and represent zone of inhibition (diameter of zone minus diameter of bacterial growth) expressed as a percentage compared to that of the wild-type nisin producer at 100%. S.D.: Standard Deviation; Relative Standard Deviation<10% for each given value. All values in bold reached statistical significance compared to nisin control (K) (Student's t-test: P<0.05). Strains marked with an asterisk are drug resistant isolates.

### Purification of nisin K12A, K12S, K12Dha, K12T and K12Dhb

The fact that the bioactivity of the K12A, K12S and K12T producers is generally not improved against strains of *L. monocytogenes* suggested that their superior bioactivity against other targets was most likely a consequence of enhanced specific activity against these targets, rather than a general enhancement arising from increased production and/or diffusion. To confirm this, nisin K12A, K12S and K12T peptides were purified by RP-HPLC to allow quantification of their specific activity by MIC determination.

**Figure 2:**
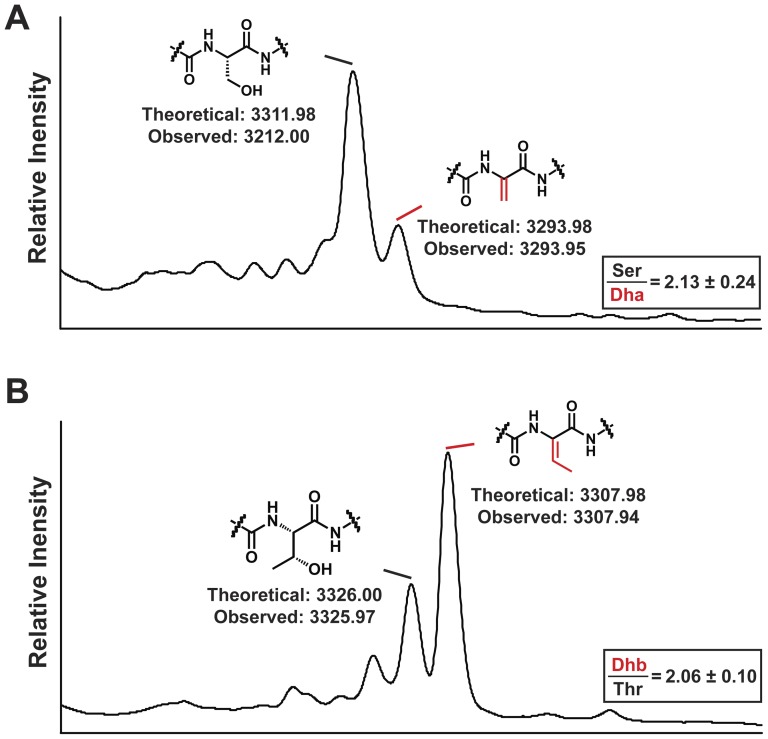
RP-HPLC Separation of nisin A K12S/Dha and K12T/Dhb. Representative RP-HPLC peaks showing the appearance of A K12S and B K12T after optimization of solvent gradient. Relative production levels were determined using triplicate peak areas from two independent purifications from first principles. Relative Standard Deviation<12% for each given value.

Nisin K12A was purified by the RP-HPLC protocol routinely employed to purify nisin and was found to be produced at similar levels as nisin A is produced by the wild-type strain (data not shown). The K12A peptide was stable during purification, lyophilisation and subsequent storage as determined by MS (data not shown). In the case of K12S and K12T, the standard RP-HPLC protocol yielded a single peak which, on the basis of MS, contained a mixture of K12S/Dha or K12T/Dhb as expected (data not shown). The production levels of K12S/Dha and K12T/Dhb were comparable to that of the wild-type producer (data not shown). Following optimisation of the solvent gradient, the two forms were successfully separated (Fig. 2). Interestingly, the degree of modification depends on the identity of the newly-incorporated residue, in that the ratio of K12S produced relative to K12Dha was approximately 2∶1, while that of K12T to K12Dhb was approximately 1∶2 (Fig. 2). Unfortunately, MS indicated that the K12S, K12Dha, K12T and K12Dhb peptides were susceptible to rapid oxidation (characteristic +16 kDa mass observed; data not shown), despite the use of a variety of strategies designed to minimise this phenomenon.

### Specific activity determination of nisin K12A

In the absence of completely unoxidised peptides, the partially oxidised K12S, K12Dha, K12T and K12Dhb peptides were employed for subsequent MIC determination. In each instance specific activity was not enhanced relative to that of nisin against *L. lactis* HP (data not shown), which is not surprising given that oxidation of nisin is known to affect its antimicrobial activity [42].

In contrast, MIC determination assays established that K12A displayed a four-fold enhanced potency against *L. lactis* HP (K12A, 15.6 nM; wild-type, 62.5 nM; Table 5). As a consequence of the significantly greater bioactivity of the K12A producer against a broad range of targets, the relative ease with which the K12A peptide was purified, and the establishment that specific activity against HP was enhanced, nisin K12A was selected for further investigation. Its specific activity was assessed against a larger selection of targets, including representative strains of *Enterococcus*, *Streptococcus*, *Bacillus* and *Staphylococcus*, as well as an additional *Lactococcus* strain. In all cases, K12A was found to be 2–4 fold more active than nisin (Table 5).

**Table 5:** Minimum inhibitory concentrations of purified nisin (WT) and nisin K12A against various Gram-positive targets.

Strain	NisinA mg/L (nM)	NisinK12A mg/L (nM)	Fold Difference
*E. faecalis* 5152	25.15 (7500)	12.36 (3750)	2
*E. casseliflavus* 5053	12.57 (3750)	6.18 (1875)	2
*E. faecium* VRE EC 533	2.10 (625)	1.03 (312.5)	2
*S. pyogenes* DSM 2071	4.19 (1250)	2.06 (625)	2
*S. mitis* UCC 5001	8.38 (2500)	4.12 (1250)	2
*B. cereus* NCIMB 700578	8.38 (2500)	2.06 (625)	4
*L. lactis* HP	0.21 (62.5)	0.05 (15.6)	4
*L. lactis* MG 1363	0.42 (125)	0.21 (62.5)	2
*S. aureus* MRSA ST 534	1.05 (312.5)	0.52 (156.3)	2

Results from minimum inhibitory concentration assays of purified nisin (WT) and nisin K12A against various Gram-positive targets. Values given are identical results from three independent determinations. Fold Difference represents the improvement of K12A compared to nisin against the relevant indicator.

Although the activity of nisin against the majority of Gram-negative bacteria is low in the absence of other stress factors such as chelating agents [43], previous mutational studies have identified specific derivatives with enhanced anti-Gram-negative activity [32], [39]. Here, an agarose-based well diffusion assay was employed [39] in order to compare the bioactivity of purified nisin and K12A against three Gram-negative strains. The results showed that the bioactivity of K12A against *E. coli* 0127∶H6 was similar to that of nisin, but that the activity of the K12A peptide was reduced against *Cronobacter sakazakii* DPC6440 and *Salmonella enterica* serovar Typhimurium UK1 (Table 6).

**Table 6:** Bioactivity of purified nisin (WT) and nisin K12A against representative Gram-negative targets.

Strain	NisinA	K12A	
	mm (S.D)	mm (S.D)	% (S.D)
*Escherichia coli* 0127∶H6	5.20 (0.43)	4.93 (0.51)	95 (4)
*Cronobacter sakazaki* DPC 6440	5.45 (0.24)	3.55 (0.17)	65 (3)
*Salmonella enterica* serovar Typhimurium UK1	4.20 (0.17)	0.77 (0.05)	18 (1)

Results from agarose gel diffusion assays of purified nisin and nisin K12A at a concentration of 40 µM against three Gram-negative strains. Results are expressed as both zone diameter and as K12A bioactivity compared to that of nisin A at 100%. Values represent the mean of triplicate agarose assay results. Standard deviation values in brackets; Relative Standard Deviation<10% for each given value. Values in bold reached statistical significance compared to nisin control (Student's t-test: P<3E^−04^).

## Discussion

In this study, a combination of site-saturation and site-directed mutagenesis was employed to generate a bank of nisin A derivatives in which K12 was substituted with all other standard amino acids. In addition to identifying promising derivatives (namely K12A, K12S, K12T, K12P), determination of the bioactivity of the K12X bank against *L. lactis* HP provided an indication of the tolerance of this position to substitution (Table 3). Overall, the K12 position of nisin was found to be very amenable to change, which is somewhat unexpected given that a lysine at this position is conserved across the six known natural nisin variants. The consequences of K12 mutagenesis can be grouped according to the nature of the newly incorporated residue. Until recently, the detrimental effects of introducing negatively charged residues into the cationic nisin [32], [37] were attributed to the importance of positive charge in the initial attraction of many cationic peptides to the cell envelope. However, the identification of the enhanced derivatives S29D and S29E demonstrated that the outcome varies depending on the location at which substitution occurs [39]. Indeed, the K12E and K12D derivatives had no detectable bioactivity, with the production of K12D being significantly diminished, while K12E is produced but is inactive (Table 3). The incorporation of positively charged residues into nisin has also had variable results [32], [37], [39], [44]. Here, K12H and K12R retained 75% and 61% activity, respectively (Table 3). Thus, the effect of charge manipulation in nisin is difficult to predict. The generally negative impact of introducing tyrosine, tryptophan or phenylalanine at position K12 is also consistent with previous reports regarding the incorporation of aromatic residues into nisin [32], [37], [39], [45], [46] (Table 3). The incorporation of the small hydrophobic residue glycine significantly decreased bioactivity of the resultant derivative, while the remaining derivatives had bioactivity comparable or improved relative to that of the wild-type (Table 3). We noted that while the inclusion of additional cysteine residues usually impacts negatively on lantibiotic production and activity [37], [39], [47], in the case of K12C bioactivity was comparable to that of the control (Table 3).

It should be noted that K12 has been the subject of previous mutational analysis in both nisin A and nisin Z (nisin Z differs from nisin A by the presence of an asparagine residue instead of a histidine at position 27). The nisin Z K12P derivative was reported to display antimicrobial properties that were similar to those of nisin Z [48], but the nisin A K12P producer described in this study had increased bioactivity relative to the corresponding wild-type peptide against *L. lactis* HP (Table 3). In the current study, the K12L producer displayed a level of bioactivity comparable to that of the wild-type (Table 3) and a previous study indicated that this mutation does not affect the specific activity of nisin A [49]. A nisin Z K12S derivative was also previously created, and, as was the case with nisin A K12S (Fig. 2), the larger portion of nisin Z K12S remained unmodified. No impact on the antimicrobial activity of nisin Z K12S was reported but, in that instance, bioactivity was assessed against only one strain [50].

Our initial screen highlighted the marked enhancement in bioactivity of the producers of nisin K12A, K12S and K12T against *L. lactis* HP. This observation prompted further analysis using a broad panel of Gram-positive microorganisms which revealed superior bioactivity against numerous pathogens, including *S. aureus* DPC5247 (a strain associated with bovine mastitis), *S. pyogenes* DSM 11728 (a pharyngitis-associated clinical isolate), *S. agalactiae* ATCC13813 (associated with early perinatal human infections and bovine mastitis), as well as numerous drug resistant strains (Table 4). The enhanced bioactivity of the K12A, K12S and K12T producers did not extend to strains of *L. monocytogenes*, with the exception of one mutant strain, EGD-e Δ*virR* [51] (Table 4). The VirRS two-component system contributes to the regulation of a number of loci that play a role in innate nisin resistance through the alteration of cell envelope charge [51], [52], [53], [54]. It is thus unsurprising that mutation of *virR*, which encodes the response regulator of this system, results in a strain that is greatly sensitised to nisin [55]. The reason for the significant bioactivity improvement of K12A, K12S and K12T relative to the wild-type against *L. monocytogenes* EGD-e Δ*virR* will be the subject of further investigation. The strain variable nature of the enhanced bioactivity of the individual derivatives provides further evidence for the well-known phenomenon that nisin derivatives can be generated with distinct target specificities.

Purification of K12S and K12T to enable specific activity studies revealed that approximately twice as much K12S as K12Dha was produced, whereas twice as much K12Dhb as K12T was made (Fig. 2). While these hydroxyl residues are incorporated at a non-native position, this observation is consistent with previous suggestions that threonines in lantibiotics are more frequently dehydrated than serines [56]. Interestingly, two relatively new and highly potent members of the extended nisin group, microbisporicin [57] and planosporicin [58], both possess a threonine in place of lysine at position 12. It is also noteworthy that the replacement of a natural lysine at position 22 with hydroxyl residues (K22T, K22S) also resulted in enhanced antimicrobial activity but, unlike the case of K12S and K12T, neither of the newly incorporated residues were modified to any detectable extent [37]. Unfortunately, nisin K12S, K12Dha, K12T and K12Dhb were found to be particularly susceptible to oxidation, thereby impeding accurate MIC determinations and preventing an assessment of the relative contribution of the individual peptides to the enhanced bioactivity of the K12S/Dha and K12T/Dhb producers.

Ultimately, the K12A producer became the focus of further study by MIC analysis utilising a variety of food-associated and clinically significant strains. These assays revealed that K12A displays a two- and four-fold improvement in specific activity against the food-grade *L. lactis* strains MG1363 and HP, respectively (Table 5). The two-fold improvement of K12A against the *Enterococcus* isolates tested, including one VRE strain, is particularly significant given that enterococci are ubiquitous in the environment [59] and can cause nosocomial infections [60]. The efficacy of K12A was improved two-fold against two clinical *Streptococcus* isolates, including *S. pyogenes* DSM 2071 (Table 5), a causative agent of streptococcal pharyngitis and its more serious sequelae. Notably, K12A was also two-fold enhanced against a strain of MRSA (Table 5). Finally, K12A showed a four-fold increase in potency against a *B. cereus* strain (Table 5), a microorganism that is widespread in soil and in foods and can cause an emetic or a diarrhoeal type of food-associated illness [61]. Thus, MIC assays determined that the molecular basis for the enhanced bioactivity of the nisin K12A producer is due to improved specific activity of the mutant peptide against the Gram-positive indicator strains tested. An agarose-based well diffusion assay with purified peptide determined that the enhanced spectrum of activity of K12A does not extend to include Gram-negative bacteria (Table 6). Once again, these results highlight the strain-variable nature of the improved activity possessed by many nisin derivatives.

In conclusion, it is apparent that altering the K12 residue of nisin A can generate derivatives with enhanced antimicrobial activity. K12A has increased potency towards numerous food-associated strains and the similar production levels of K12A to nisin A suggests that the standard industrial nisin purification/fermentation methods could be utilized. However, further studies will be needed to investigate the physical and chemical properties of K12A before it can be considered for use as a biopreservative, including its solubility and stability at different temperatures and varying pH. From a veterinary perspective, K12A appears to have potential with respect to the treatment of bacteria that are responsible for bovine mastitis (staphylococci and streptococci), as well as showing promise as a novel candidate for treatment of *S. pyogenes* infection. There is a pressing need for the development of novel antimicrobials, especially given that infections now occur that are resistant to all current antibacterial treatments [62]. The improved specific activity of K12A towards MRSA ST528 and VRE EC533 suggests that K12A merits further investigation with respect to its application against such antibiotic resistant targets. Future work will also focus on the elucidation of the mechanistic basis for the enhanced activity of K12A relative to nisin, as well as determining the effect of combining the K12A mutation with other substitutions previously found to increase the potency of nisin, alter its physico-chemical properties, or modify its activity spectrum.
